# Factors Associated with Insulin Resistance in a Middle-Aged Non-Obese Rural Population: The Chungju Metabolic Disease Cohort (CMC) Study

**DOI:** 10.4178/epih/e2011009

**Published:** 2011-09-26

**Authors:** Sun Young Lim, Hee Sung Ha, Hyuk-Sang Kwon, Jin-Hee Lee, Hyeon Woo Yim, Kun-Ho Yoon, Won-Chul Lee, Ho-Young Son, Yong-Moon Park

**Affiliations:** 1 Graduate School of Public Health, The Catholic University of Korea, Seoul, Korea.; 2 The Catholic Institute of Ubiquitous Healthcare, The Catholic University of Korea, Seoul, Korea.; 3 Department of Preventive Medicine, College of Medicine, The Catholic University of Korea, Seoul, Korea.; 4 Division of Endocrinology and Metabolism, Department of Internal Medicine, College of Medicine, The Catholic University of Korea, Seoul, Korea.

**Keywords:** Abdominal obesity, BMI, Insulin resistance

## Abstract

**OBJECTIVES:**

We aimed to determine the characteristics affecting insulin resistance in non-obese middle-aged adults in a rural community.

**METHODS:**

A total of 1,270 non-diabetic adults aged between 40 and 64 years old with body mass index (BMI) less than 25 kg/m^2^ were analyzed. Subjects with insulin resistance were defined as those who had the highest quartile value of the homeostasis model assessment of insulin resistance (HOMA-IR) in a non-diabetic population.

**RESULTS:**

A total of 217 subjects (20.6%) had insulin resistance. Prevalence of metabolic syndrome was significantly higher in insulin-resistant subjects in both men (29.3% vs. 10.3%) and women (34.1% vs. 15.6%). Among metabolic syndrome components, elevated waist circumference and elevated triglyceride were higher in insulin-resistant subjects in both genders. After being controlled for socioeconomic status and lifestyle related covariates, the association between insulin resistance and BMI was statistically significant in the category of 23.0-24.9 kg/m^2^ in men (adjusted OR, 4.63; 95% confidence interval [95% CI], 1.77-12.15) using the category of 18.5-20.9 kg/m^2^ as a reference. In addition, the association between insulin resistance and abdominal obesity was statistically significant only for men (adjusted OR, 2.57; 95% CI, 1.29-5.11).

**CONCLUSION:**

Insulin resistance appears to be highly associated with high BMI and abdominal obesity, even in non-obese, non-diabetic middle-aged men.

## INTRODUCTION

Obesity is a risk factor for various types of metabolic disease including type 2 diabetes and hypertension [1-3], and it has been considered as a major health issue in many countries [4]. One of the diagnostic indicators for obesity, body mass index (BMI), has a simplified measurement method. In addition, it has been known as a useful tool for predicting risks of developing various types of cardiovascular disease [5]. However, a BMI-based diagnosis of obesity cannot include all the scope of obesity. For instance, in all the patients who were diagnosed as obese, there was a lack of the collection of metabolic risk factors associated with cardiovascular disease. It has also been reported that the people in the non-obesity range did not always show healthy metabolic status [6].

Of these concepts, patients who were not apparently obese, i.e., had a normal profile based on BMI criteria but had obesity-related metabolic abnormalities, were defined as "Metabolically Obese Normal Weight (MONW)" by Ruderman et al. [7]. It was hypothesized that these subjects showed hyperinsulinemia or insulin resistance, acquired a metabolically obese status and had a higher degree of risk of developing cardiovascular disease [8]. In the MONW group, as compared with the control group, the incidence of abdominal obesity and visceral fat was relatively higher. With the presentation of hypercholesterolemia, low high-density lipoproteinemia, hypertriglyceridemia and high blood pressure, risks of developing cardiovascular disease have been reported to be relatively higher [9,10]. In addition, a prospective cohort study shows that higher risks of developing type 2 diabetes and cardiovascular disease have been found in the MONW group as compared with subjects who were metabolically healthy. These results were also shown to be relatively higher following a comparison with obese subjects who were metabolically healthy [11].

Through the active identification, with the use of such interventions as diet, exercise therapy and drug treatments, the necessity for the delay of disease progression has been proposed [12]. For the appropriate management of these patients, their characteristics should first be clarified. In Korea, however, there are few studies about these issues. Other than small sample sizes in most of the pre-existing studies, there were also limitations that socioeconomic status and lifestyle-related characteristics were not considered.

The purpose of this study was to determine the characteristics of clinical, socioeconomic, and lifestyle features affecting the insulin resistance in non-obese middle-aged adults who were residing in a rural community.

## MATERIALS AND METHODS

### Subjects

The current study was conducted in adults aged 40 years or older who were residing in rural districts of Chungju area between 2005 and 2006 from February to April in each year. Subject areas were stratified into 29 rural health subcenters and health care posts. Using a stratified random cluster sampling in which towns belonging to each district were classified as a cluster, 166 towns were selected and 6,388 subjects participated in the current study [13-15]. Of them, based on the BMI, 1,679 non-obese (18.5-24.9 kg/m^2^) adults aged between 40 and 64 years (males: 761 and females: 918) were enrolled in the current study. After excluding 409 subjects (those with a past history of cardiovascular diseases, diabetes mellitus or hypertension, and those whose fasting glucose and fasting insulin were dropped out), 1,270 subjects (males: 598 and females: 672) were finally selected as a study population. The current study was approved by The Institutional Review Board (IRB) of The Catholic University of Korea, the Kangnam St. Mary's Hospital.

### Measurements

A questionnaire survey was performed to examine lifestyle-related characteristics such as smoking, drinking and exercise habit. The status of smoking and drinking alcohol were examined. The type of exercise, the mean frequency of exercise and exercise time were examined. Sociodemographic variables include the educational status, occupation, the total monthly income, and living with spouse of the subjects. Eating over 20 kinds of the food items including meat, fish, vegetable, fruit and rice in a day was defined as a balanced diet [16].

Using the standardized methods, the height, weight, and waist and the hip were measured. The height, waist and hip were measured at a unit of 0.1 cm. The weight was measured at a unit of 0.1 kg. BMI was calculated using the formula weight (kg)/height (m^2^). The systolic blood pressure and diastolic blood pressure were measured using a sphygmomanometer in a sitting position after a more than 10-minute stabilization prior to blood sampling. In the right upper arm, the blood pressure was measured twice. Then, in cases in which the difference in the diastolic pressures was smaller than 5 mmHg, the average value of two measurements was obtained.

Prior to blood sampling, all subjects were fasted over 12 hours and blood analysis was performed in a central laboratory. Total serum cholesterol and triglyceride were measured using an enzymatic calorimetric test. High-density lipoprotein (HDL) cholesterol was measured using a selective inhibition method, and low-density lipoprotein (LDL) cholesterol was calculated using the Friedewald formula [17]. Following the blood sampling, a sodium fluoride tube was used to store and transfer fasting glucose. It was measured using the hexokinase method. Fasting serum insulin was measured using a Radiommunoassay kit (Dainabot, Japan).

In the current study, of those whose BMI was within a normal range (18.5-24.9 kg/m^2^), to define the group where there was a insulin resistance, insulin resistance was assessed using the homeostasis model assessment of insulin resistance (HOMA-IR). Of 6,388 adults aged 40 years or older who participated in the current study, excluding patients with diabetes mellitus, 5,779 were used to establish a critical point which corresponds to the highest quartile number of HOMA-IR in each year (2005, 1.69; 2006, 1.74). Of subjects who were enrolled in the current study, those who corresponded to more than a critical point were considered to have an insulin resistance. The formula for calculating HOMA-IR was as follows:

(HOMA-IR=fasting insulin (µU/mL)×fasting glucose (mmol/L)/22.5) [18].

A definition of metabolic syndrome was made based on the criteria of modified NCEP-ATP III (Third report of National Cholesterol Education Program Expert panel on detection, evaluation and treatment of high blood cholesterol in adults-Adults Treatment Panel III, 2001) [19] using the criteria of abdominal obesity based on the Korean Society for the Study of Obesity diagnostic criteria of male ≥90 cm and female ≥85 cm [20].

### Statistical analysis

To compare the characteristics between insulin-resistant subjects and the other group by gender, a chi-square test for categorical variables and student's t-test for continuous variables were applied. For parameters showing non-normal distributions (triglycerides, fasting glucose, fasting insulin, and HOMA-IR), Wilcoxon rank sum test was performed. To examine the factors associated with insulin resistance in the non-obese group, a multiple logistic regression was performed by gender. Variables of p-value <0.25 in the univariate test were selected as candidates for the multivariate model along with age. All two-tailed p-values of 0.05 were regarded as indicating statistical significance. All the statistical analyses were performed using SAS version 9.1 (SAS Inc., Cary, NC, USA).

## RESULTS

### A comparison of the general and lifestyle-related characteristics between the subjects with insulin resistance and without insulin resistance

A total of 82 men (13.7%) and 135 women (20.1%) were insulin-resistant subjects. The proportion of insulin-resistant subjects was higher in subjects with over nine years of education (p=0.042) for men, and lower in farmers for women (p=0.041) (Table 1).

### A comparison of clinical characteristics between the subjects with insulin resistance and without insulin resistance

BMI, waist circumference, total cholesterol, total cholesterol to high-density lipoprotein cholesterol ratio, fasting glucose, fasting insulin, and HOMA-IR were significantly higher in insulin-resistant subjects for both men and women. HDL cholesterol was significantly lower in insulin-resistant subjects for both men and women. Triglyceride, LDL cholesterol and triglyceride to HDL cholesterol ratio were significantly higher in insulin-resistant subjects only for women (Table 2).

### A comparison of metabolic syndrome and its components between insulin-resistant subjects and others by gender

A comparison was made for the prevalence of metabolic syndrome and its components (modified NCEP-ATP III criteria) by gender. The prevalence of metabolic syndrome was about three times higher for men and over two times higher for women in the insulin-resistant subjects compared to the non-insulin-resistant subjects. Of various categories of metabolic syndrome, high waist circumference, high triglycerides, and high fasting plasma glucose showed a significantly higher prevalence in the insulin-resistant subjects in both genders (Table 3).

### Factors associated with insulin resistance in the non-obese population

After being controlled for all other covariates, the association between insulin resistance and BMI was statistically significant in the category of 23.0-24.9 kg/m^2^ in men (adjusted OR, 4.63; 95% confidence interval [95% CI], 1.77-12.15) using the category of 18.5-20.9 kg/m^2^ as a reference. The association between insulin resistance and abdominal obesity was statistically significant only for men (adjusted OR, 2.57; 95% CI, 1.29-5.11) (Table 4).

## DISCUSSION

In the present study, we demonstrated that high BMI and abdominal obesity were associated with insulin resistance in non-obese middle-aged men living in rural areas. Insulin resistance was also associated with higher prevalence of metabolic syndrome and its components such as elevated waist circumference and elevated triglyceride in both genders. It is well known that insulin resistance increases the risk for type 2 diabetes and cardiovascular disease, even for those within the normal BMI range [11]. This finding could be more significant in Asians who are generally less obese but have more body fat than Caucasians, which renders them to have more obesity-related risks for cardiovascular diseases with similar BMI [21].

Waist circumference was found to be significantly higher in insulin-resistant subjects not only according to the criteria of abdominal obesity based on the Korean Society for the Study of Obesity, but also the criteria of abdominal obesity based on the Asia-Pacific diagnostic criteria of male ≥90 cm and female ≥80 cm [22]. Central obesity has been mentioned as one of the major characteristics observed in the MONW group [12], and it has also been known to be correlated with insulin resistance [23,24]. The relationship between central obesity and insulin resistance could be explained based on the metabolism of free fatty acids or other adipocytokines (TNF-α, IL-6, adiponectin) released from the visceral fat [25]. The abdominal obesity and visceral fat were reportedly increased in insulin-resistant subjects [9].

We noted that high BMI and abdominal obesity were associated with insulin resistance only in non-obese men. This gender-specific discrepancy in the development of metabolic abnormalities has been explained by sex differences in associations between inflammatory markers [26] and endogenous sex hormones [27] based on the data from Caucasian studies. However, well-designed prospective cohort studies are warranted to elucidate this phenomenon in non-obese Asians.

In this study, we found that metabolic syndrome, a potent risk factor for cardiovascular disease [28], had a prevalence that was more than two times higher in insulin-resistant subjects. In addition to elevated waist circumference, it was typically observed that elevated triglyceride was significantly higher in insulin-resistant subjects in both genders. In the previous studies conducted in western countries [29,30] cholesterol level was significantly higher in insulin-resistant subjects. On the other hand, a study of non-diabetic Korean adults shows that insulin resistance was associated with high triglycerides [31]. In addition, a Japanese study shows that triglyceride level was uniquely higher in insulin-resistant subjects, suggesting that the increased level of triglyceride would play a role in association with insulin resistance in the non-obese Asian people [10].

In the current study, to define insulin-resistant subjects, an insulin resistance was assessed with the application of HOMA-IR. This was used to presume the functions of beta cells as well as insulin sensitivity through fasting insulin and fasting glucose. It is also known to be highly correlated with insulin resistance measured with the hyperinsulinaemic clamp method [18]. Accordingly, it has widely been used as a tool for measuring insulin resistance in a large-scale community-based epidemiological study. Other methods to define the insulin resistance include measuring visceral fat areas [32,33] and using NCEP-ATP III metabolic syndrome diagnostic criteria [11,34]. Despite the differences in defining insulin resistance observed in each study, in regard to the characteristics of insulin resistant subjects, consistent results have been mostly proposed [9].

The current study has limitations that should be considered. The areas of visceral fat were not measured although they were a valid estimator of the abdominal obesity, which was mentioned as one of the major characteristics of the non-obese group with an insulin resistance. Due to a cross-sectional design, the current study has difficulty determining the causality in regard to the observed relationship between the factors and insulin resistance. In such a condition that there are few Korean studies, however, the results of the current study could be considered to be significant.

In conclusion, in middle-aged adults who were apparently non-obese, insulin resistance defined by highest quartile of HOMA-IR was associated with high BMI and abdominal obesity for men. Further prospective studies are warranted to clarify risk factors, which is essential for detecting insulin resistant subjects and performing the appropriate interventions for them.

## Figures and Tables

**Table 1:**
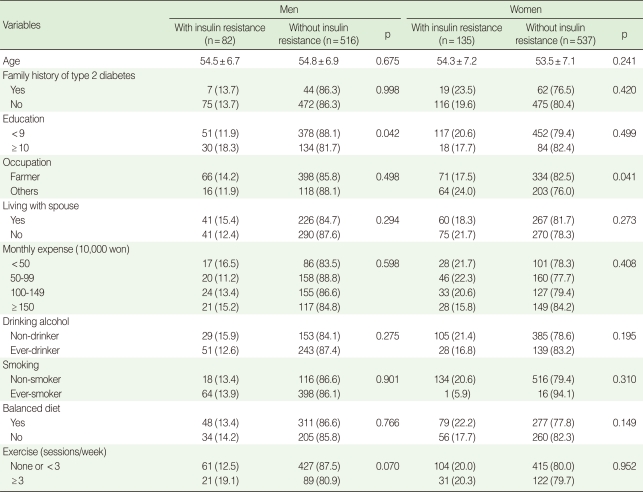
General and lifestyle-related characteristics of the subjects with insulin resistance and without insulin resistance by gender

Data are summarized as a mean±SD or n (%).

**Table 2:**
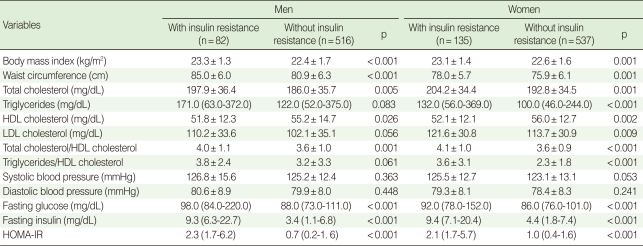
Clinical characteristics of the subjects with insulin resistance and without insulin resistance by gender

Data are summarized as a mean±SD, a median (5-95%) or n (%). HDL high-density lipoprotein; LDL, low-density lipoprotein; HOMA-IR, homeostasis model assessment of insulin resistance.

**Table 3:**
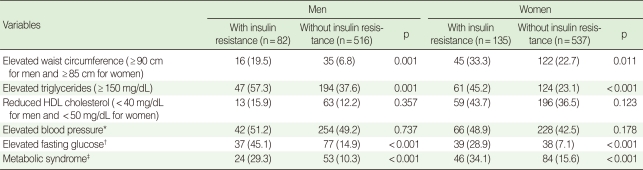
Comparison of metabolic syndrome and its components between the subjects with insulin resistance and without insulin resistance by gender

Data are summarized as N (%). HDL, high-density lipoprotein. ^*^Systolic blood pressure ≥130 mmHg or diastolic blood pressure ≥85 mmHg or antihypertensive medication of previously diagnosed hypertension; ^†^Fasting plasma glucose ≥100 mg/dL or antidiabetic treatment; ^‡^modified NCEP-ATP III definition.

**Table 4:**
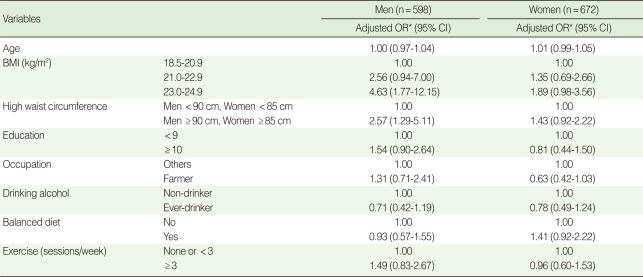
Factors associated with insulin resistance by gender

BMI, body mass index; OR, odds ratio; CI, confidence interval. ^*^Odds ratio adjusted by all other covariates in each variable.
